# Retraction Note: Modulation of aberrant CDK5 signaling rescues impaired neurogenesis in models of Alzheimer’s disease

**DOI:** 10.1038/s41419-026-09413-6

**Published:** 2026-11-06

**Authors:** L. Crews, C. Patrick, A. Adame, E. Rockenstein, E. Masliah

**Affiliations:** 1 Department of Neurosciences, University of California, San Diego, La Jolla, CA USA; 2 Department of Pathology, University of California, San Diego, La Jolla, CA USA

Retraction Note to: *Cell Death & Disease* https://doi.org/10.1038/cddis.2011.2, published online 10 February 2011

The Editors have retracted this article. After publication, concerns were raised regarding some of the data presented in the figures and supplementary materials, specifically:

- In Fig. 4j–l, the merged image (l) doesn’t fully match the DCX (j, green) and CDK5 (k, red) channels, and there appears to be a duplicated pattern in the top left of the merged image;
- Fig. 8 panels f and g appear to overlap;
- Fig. S4 panels f and g appear to have highly similar artifacts (with different brightness), and Fig. S4g appears to show a plain black area with no background signal.

The Editors therefore no longer have confidence in the presented data.

L Crews and E Masliah do not agree with this retraction. E Rockenstein is deceased. C Patrick and A Adame have not responded to any correspondence from the editor or publisher about this retraction.

